# Image Reconstruction from Sparse Low-Dose CT Data via Score Matching

**Published:** 2023-06-23

**Authors:** Wenxiang Cong, Wenjun Xia, Ge Wang

**Affiliations:** Biomedical Imaging Center, Department of Biomedical Engineering, Rensselaer Polytechnic Institute, Troy, NY 12180

**Keywords:** Computed tomography (CT), radiation dose reduction, sparse-view image reconstruction, maximum a posteriori (MAP) estimation, machine learning, diffusion model, score function

## Abstract

Computed tomography (CT) reconstructs sectional images from X-ray projections acquired from multiple angles around an object. By measuring only a fraction of full projection data, CT image reconstruction can reduce both radiation dose and scan time. However, with a classic analytic algorithm, the reconstruction of insufficient data CT always compromises structural details and suffers from severe artifacts. To address this issue, we present a deep learning-based image reconstruction method derived from maximum a posteriori (MAP) estimation. In the Bayesian statistics framework, the gradient of logarithmic probability density distribution of the image, i.e., the score function, plays a crucial role, contributing to the process of image reconstruction. The reconstruction algorithm theoretically guarantees the convergence of the iterative process. Our numerical results also show that this method produces decent sparseview CT images.

## 1. Introduction

X-ray computed tomography (CT) is a crucial imaging modality used in medical, security, and industrial applications [1]. Filtered back-projection (FBP) is a classic image reconstruction method for CT [2]. In the cases of low-dose and/or sparse-view CT scans, FBP generates image noise and artifacts in reconstructed images [3–5]. To address this challenge, iterative methods were developed to incorporate prior knowledge of images and imaging system. These methods model a statistical distribution of photons to improve low-dose CT image quality [5, 6]. Iterative algorithms can also incorporate compressed sensing (CS) to enforce the sparsity of reconstructed images for improving image quality. Total variation (TV) minimization is a common regularization method for the image reconstruction [4,6]. However, the TV method often oversmoothes textured regions and eliminates subtle details. The low-dimensional manifold model (LDMM) interprets image patches as a point cloud sampled from a low-dimensional manifold embedded in a high-dimensional ambient space, providing a new way for regularization by minimizing the dimensionality of the corresponding image patch manifold [7]. The patch manifold of images typically exhibits a low-dimensional structure and yet accommodates rich features [8]. With the LDMM prior knowledge on images, the iterative reconstruction method would lead to improve image quality [7].

Emerging deep learning is a powerful technique for various tasks through learning and inference in a data-driven fashion [9]. These learning techniques can perform various types of uncertainty estimation and data modeling. In this paper, we develop a deep-learning-based image reconstruction method for sparse low-dose CT data. We model the X-ray projection data as a Poisson distribution, enabling us to reconstruct the image using maximum a posteriori (MAP) estimation. In the Bayesian statistics framework, the gradient of logarithmic probability density distribution of the image plays a crucial role, contributing to the process of image reconstruction. A score function is defined as the gradient of the logarithmic density a probability distribution, and can be modeled by parameterizing a deep neural network. It is underlined that the score function in principle models the density distribution of underlying images, which differs significantly from traditional regularization functions in iterative reconstruction algorithms such as TV minimization. The score function essentially preserves the same amount of information as the associated probability density function, representing a promising research avenue towards better data modeling, generation and inference. Therefore, this data-driven method can obtain optimal prior knowledge of the image probability density distribution for CT image reconstruction. The rest of this paper is organized as follows. In Section II, the methodology is described, especially presenting a neural network to learn the score function of an underlying image distribution from a training dataset to generate high-quality samples and evaluate their probability distribution. Then, the statistical iterative procedure with score function is performed for the CT image reconstruction. The convergence of the iterative sequence is proven through rigorous mathematical analysis. In Section III, representative image reconstruction results are reported in numerical simulation, demonstrating the feasibility of the proposed score matchingbased image reconstruction method. Additionally, our results are favorably compared with the well-known techniques such as the simultaneous algebraic reconstruction technique (SART), as well as advanced methods like SART with total variation (TV) regularization and SART with low-dimensional manifold model (LDMM) regularization. Finally, in Section IV, relevant issues are discussed.

## 2. Methodology for Low-dose Sparse CT

### 2.1. Bayesian Framework

In X-ray imaging, the number of X-ray photons recorded by a detector element is a random variable ξ, which can be modeled as the Poisson distribution [2]:

(1) $$p\left(\xi =y_{i}\right)=\frac{{\left({\overset{‾}{y}}_{i}\right)}^{y_{i}}}{y_{i}!}\text{exp}\left(-{\overset{‾}{y}}_{i}\right),$$

where ${\overset{‾}{y}}_{i}$ is the expectation value of recorded X-ray photons along a path l from the X-ray source to the $i^{\text{th }}$ detector element, and obeys the Beer-Lambert law:

(2) $${\overset{‾}{y}}_{i}=n_{i}\text{exp}\left(-\int_{l}\;\;\mu (\vec{r})dl\right),$$

where $n_{i}$ is the number of X-ray photons recorded by the $i^{\text{th }}$ detector element in the blank scan (without any object in the beam path), and μ(r) is the linear attenuation coefficient distribution within an object to be reconstructed. For numerical implementation, Eq. (2) is discretized as,

(3) $${\overset{‾}{y}}_{i}=n_{i}\text{exp}\left(-A_{i}\mu \right),$$

where μ is a vector of pixel values in the linear attenuation coefficient image, and $A_{i}$ is weighting coefficients of the pixel values along the $i^{\text{th }}$ beam path. Assuming that measurements are independent, the likelihood function of the X-ray projection data can be obtained as

(4) $$p(Y|\mu )=\prod_{i=1}^{m}\;\frac{{\left({\overset{‾}{y}}_{i}\right)}^{y_{i}}}{y_{i}!}\text{exp}\left(-{\overset{‾}{y}}_{i}\right),$$

where $Y={\left(y_{1},y_{2},\cdots ,y_{m}\right)}^{T}$ is the number of photons measured by detectors, and m is the total number of X-ray detectors. Based on the Bayesian theorem: p(μ∣Y)p(Y)=p(Y∣μ)p(μ), the image reconstruction can be performed using the maximum a posteriori (MAP) estimation, which is equivalent to the following minimization problem:

(5) $$\mu_{\text{min}}=\underset{\mu }{\text{arg}\text{min}}\left(\sum_{i=1}^{m}\;\;\left[{\overset{‾}{y}}_{i}-y_{i}\text{log}\left({\overset{‾}{y}}_{i}\right)\right]-\text{log}p(\mu )\right),$$

where log⁡(p(μ)) is a logarithmic probability density of an attenuation image expressing the prior knowledge about images μ in a specific application domain. Combining Eqs. (3)–(5), we have

(6) $$\mu_{\text{min}}=\underset{\mu }{\text{arg}\text{min}}\left(\sum_{i=1}^{m}\;\;\left[n_{i}\text{exp}\left(-A_{i}\mu \right)+y_{i}A_{i}\mu \right]-\text{log}p(\mu )\right).$$

Applying the second-order Taylor approximation, Eq. (6) can be simplified to a quadratic optimization problem [6]:

(7) $$\mu_{\text{min}}=\underset{\mu }{\text{arg}\text{min}}\left[\frac{1}{2}(A\mu -b{)}^{T}D(A\mu -b)-\text{log}p(\mu )\right]$$

where A is the m×n system matrix composed of the row vectors $A_{1},A_{2},\cdots ,A_{m}$, and D is the diagonal matrix of the form $\text{diag}\left(y_{1},y_{2},\cdots ,y_{m}\right)$. The optimization problem defined by Eq. (7) can be solved using the gradient-based iterative method in the algebraic reconstruction technique framework:

(8) $$\mu^{k+1}=\mu^{k}-\left(\omega A^{T}D\left(A\mu^{k}-b\right)-\sigma \nabla \text{log}p\left(\mu^{k}\right)\right),\;k=1,2,\cdots$$

where ∇log⁡p(μ) is the score function of the probability density distribution with respect to the current image, and ω and σ are parameters for trade-offs between the data fidelity and sample plausibility.

### 2.2. Convergence of the Score-matching Reconstruction Scheme

For the convergent analysis of the iteration scheme (8), we assume that A is a m×n system matrix and has rank n. The probability density function p(μ) is assumed to be sufficiently smooth, and the Hessian matrix $\nabla^{2}\text{log}p(\mu )$ of the logarithmic probability density function has bounded eigenvalues [10,11], denoted by $\left|h_{i}\right|<C,\;i=1,2,\cdots ,n$. Based on these assumptions, we have the following Lemma for the convergence of the iteration scheme (8) [12].

**Lemma 1:** The iteration scheme in Eq. (8) is convergent.

**Proof:** Based on the iteration procedure Eq. (8), we obtain

(9) $$\mu^{k+1}-\mu^{k}=\left(I-\omega A^{T}DA\right)\left(\mu^{k}-\mu^{k-1}\right)+\sigma \left[\nabla \text{log}p\left(\mu^{k}\right)-\nabla \text{log}p\left(\mu^{k-1}\right)\right],$$

From the mean value theorem of a multivariate function, there exists a vector ξ such that,

(10) $$\text{log}p\left(\mu^{k}\right)-\text{log}p\left(\mu^{k-1}\right)=\nabla \text{log}p(\xi )\left(\mu^{k}-\mu^{k-1}\right)$$

From Eqs. (9–10), we obtain

(11) $$\mu^{k+1}-\mu^{k}=\left(I-\omega A^{T}DA+\sigma \nabla^{2}\text{log}p(\xi )\right)\left(\mu^{k}-\mu^{k-1}\right)$$

where $\nabla^{2}\text{log}p(\xi )$ is the Hessian matrix of the logarithmic probability density function and is symmetric. Since $(Ax{)}^{T}DAx=\sum_{i=1}^{m}\;y_{i}{\left(A_{i}\cdot x\right)}^{2}>0$ for any nonzero vector $x\in R^{n}$, the matrix $A^{T}DA$ is positive definite, denoting its smallest and largest eigenvalues as $\lambda_{\text{min}}$ and $\lambda_{\text{max}}$ respectively. From Eq. (11), it is easy to find that by choosing the parameters ω and σ to satisfy $0<\sigma <\omega r_{\text{min}}/C$ and $\sigma C/r_{\text{min}}<\omega <(2-\sigma C)/r_{\text{max}}$, there exist a positive constant 0<q<1 such that $∥\mu^{k+1}-\mu^{k}∥\leq q∥\mu^{k}-\mu^{k-1}∥$, and $∥\mu^{k+1}-\mu^{k}∥\leq q^{k}∥\mu^{1}-\mu^{0}∥$. Hence, according to the Cauchy convergence criterion, the image sequence $\left\{\mu^{k}|k=0,1,\cdots \right\}$ must be convergent.

### 2.3. Score function estimation

The score function is the gradient of the logarithmic probability density function with respect to images, i.e., ∇log⁡p(x). The unknown score function in Eq. (8) must be estimated in advance to perform for the image reconstruction. If a known dataset $\left\{x_{i}|i=1,2,\cdots ,N\right\}$ sampled from a data distribution $p_{\text{data }}(x)$ is available, the score function ∇log⁡p(x) can be estimated from image dataset based on machine learning technique [13,14]. Interestingly, Langevin dynamics equation describes the relation between samples and score function to produce samples from the score function $\nabla_{x}\text{log}p(x)$ [14]. Similarly, we can also estimate the score function from the samples in the context. The Langevin dynamic uses stochastic differential equation to describe the dynamics of molecular systems based on Newton’s second law of motion,

(12) $$\lambda \dot{x}=-\nabla E(x)+\varepsilon .$$

The energy function E(x) assigns energy values to all its possible states. The expectation value of energy function can be expressed as,

(13) $$\overset{‾}{E}=\sum_{x}\;p(x)E(x).$$

Entropy measures the amount of uncertainty represented by this probability distribution. The maximum entropy principle states that the most appropriate distribution to model a given set of data is the one with the highest entropy among all those that satisfy our prior knowledge’s constraints [15],

(14) $$\underset{p(x)}{\text{max}}\;\left(-\sum_{x}\;\;p(x)\text{log}p(x)\right).$$

The solution of the optimization problem (14) gives the probability function as,

(15) $$p(x)=\frac{\text{exp}(-\tau E(x))}{\sum_{x}\;\;\text{exp}(-\tau E(x))}.$$

From Eq. (15), the gradient of the log-probability is the negative gradient of the energy,

(16) ∇log⁡p(x)=-τ∇E(x).

Thus, Langevin equation becomes:

(17) $$\frac{dx}{dt}+\varepsilon =\frac{1}{\lambda \tau }\nabla_{x}\text{log}p(x),$$

here λ and τ are constant. The differential term on the right hand side of Eq. (17) can be simulated by following optimization,

(18) $$D_{\theta }\left(x,\sigma \right)=\text{arg}\text{min}\left(\sum_{x∼p_{\text{data }}\left(x\right)}\;\;\Sigma_{n∼\text{N}\left(0,\sigma^{2}I\right)}{∥D_{\theta }\left(x+n,\sigma \right)-x∥}_{2}^{2}\right),$$

where $x∼p_{\text{data }}(x)x$ is a training data and $n∼\text{N}\left(0,\sigma^{2}I\right)$ is the noise. Thus, we have the score function estimation,

(19) $$\nabla \text{log}p(x,\sigma )=\left(D_{\theta }(\text{x},\sigma )-x\right)/\lambda \tau \sigma^{2}.$$

## 3. EXPERIMENTAL DESIGN AND RESULTS

### 3.1. Dataset:

To evaluate the performance of our method for low-dose sparse-view CT reconstruction, we selected the NIH-AAPM-Mayo Clinic Low-Dose CT Grand Challenge dataset to perform realistic numerical simulation. The dataset has 2,378 CT images with a slice thickness of 3mm from 10 patients and was randomly divided into a training dataset and a test dataset. The training dataset contains 1,923 images from 8 patients, and the test dataset has 455 images from the remaining 2 patients. The original image size is 512×512. The distance from the X-ray source focal spot to the isocenter of the imaging field of view is 541mm. The X-ray imaging geometry was set to a distance of 949.15mm from the detector to the source. The number of detector elements is 888. The dimension of each detector element is 1.024mm. The Xray CT imaging was simulated using the distance-driven algorithm. A total of 90 projection views are uniformly distributed over a 360-degree angular range to generate a few-view projection datasets. The projection datasets were corrupted by Poisson noise to simulate real xray imaging experiments for the tomographic image reconstruction.

### 3.2. Network Training:

The standard training procedure was followed to perform the training, validation, and testing. We used the ResNet neural network to simulate the score function described in Section 2.3. The ResNet network are one residual block including three convolution layers with 64 filters of 7×7 kernels, followed by one residual block including three convolutional layers with 64 filters of 5×5 kernels, and one residual block including three convolution layers with 64 filters of 3×3 kernels. Each residual block works in a feed-forward fashion with the shortcut connection skipping three layers to implement an identity mapping. Then, one convolution layer with 64 filters of 3×3 kernels is followed by one convolution layer with 32 filters of 3×3 kernels, and the last layer generates only one feature map with a single 3 ×3 filter as the output. Every layer is followed by a ReLU unit. The ResNet network is trained using image patches of 64×64. Training the network is to find kernels in convolution layers to minimize loss function on a training dataset. The training procedure was programmed in Pytorch on a PC computer with an NVIDIA Titan XP GPU of 12 GB memory. The network parameters in the convolution kernels were randomly initialized according to the Gaussian distribution with mean of zero and variance of 0.01. The optimization was conducted using the ADAM algorithm with $\beta_{1}=0.9,\beta_{2}=0.999$. The network was trained using 1000 epochs at the learning rate of 10^−4^. The training process took about 24 hours. As a result, the training process showed excellent convergence and stability. The learned score function was used in the iterative formula (8) for the image reconstruction.

### 3.3. Image Reconstruction:

**Figure 1.**
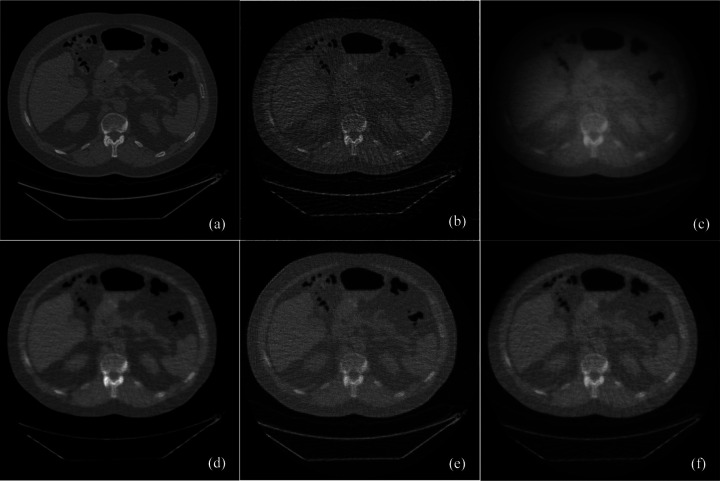
The comparison of images reconstruction between conventional and proposed methods. (a) The ground truth image, (b) the image reconstructed from 90 projections using FBP, (c) the image reconstructed from 90 projections using the SART iteration, (d) the image reconstructed from 90 projections using the score function-based iteration, (e) the image reconstructed from 90 projections using the SART iteration with low-dimensional manifold model, and (f) the image reconstructed from 90 projections using the SART iteration with total variation minimization.

Few-view image reconstructions were performed from 90 projection dataset using the standard filtered backprojection (FBP), simultaneous algebraic reconstruction technique (SART), SART with total variation (TV) regularization, SART with low-dimensional manifold model (LDMM) regularization, and the score function-based reconstruction algorithms, respectively. Figure 1 presented the images reconstructed from the score-function-based algorithm and the conventional image reconstruction methods. Clearly, the proposed score matching-based reconstruction method well preserved structural information, especially texture features, achieving excellent spatial and contrast resolution while significantly reducing image noise. The peak signal-to-noise ratio (PSNR) and structural similarity index measure (SSIM) were also used to evaluate sparse-view image reconstructions. Significantly, the proposed learning-based reconstruction method achieved average PSNR of 18.14dB and SSIM of 0.7017, which are higher than the other conventional reconstruction methods, as shown in Table 1.

## 4. Discussions and Conclusion

In this study, we model the X-ray projection data as the Poisson distribution, enabling us to reconstruct image using maximum a posteriori (MAP) estimation. The MAP estimation is to minimizing the image fidelity term while maximizing the logarithmic probability density of the image. The image reconstruction process relies on gradient-based optimization methods, which operate through an iterative procedure. The score function plays an important role in the image reconstruction by providing the gradient of the logarithmic probability density function. It incorporates statistical knowledge and serves as a key element in this iterative process, contributing to refine the reconstructed image for the each iteration.

To accurately estimate the score function, we adopt a deep convolutional neural network (CNN) to simulate the score function, which is learned from the image dataset using a score matching method. The score matching-based image reconstruction method, facilitated by the learned score function, offers notable advantages for low-dose sparse-view CT. In comparison to well-known techniques such as the simultaneous algebraic reconstruction technique (SART), as well as advanced methods like SART with total variation (TV) regularization and SART with low-dimensional manifold model (LDMM) regularization, our approach demonstrates improved image quality, which indicates that our score matching-based method surpasses these existing methods in terms of image quality metrics in low-dose sparse-view CT scenarios.

Compressed sensing is based on the assumption that images are sparse or compressible in some transform domain, such as wavelet or Fourier transform. However, this assumption may not hold for some images, particularly for complex and fine-structured biomedical images. In contrast, the score function approach provides a more flexible and data-driven prior information for image reconstruction, as it is learned directly from the data distribution. This allows for a more accurate representation of the underlying structure of the images. In conclusion, we have formulated the reconstruction of CT images via score-matching and demonstrated the feasibility and merits of this new method. The proposed score matching-based iterative method of image reconstruction has been theoretically demonstrated to converge to a minimum of the MAP objective. Moreover, the quantitative evaluation using PSNR and SSIM has shown that this method can achieve improved image quality in low-dose sparse-view CT. The proposed approach can reduce radiation dose and improve contrast resolution for the detection and characterization of lesions and other diseases. Further system evaluation and optimization studies are ongoing to explore the potential application of this new approach in CT imaging

## Figures and Tables

**Table 1.** Reconstructed image quality metric

	Score function	FBP	SART	SART+TV	SART+LDMM
SSIM	0.7017	0.3703	0.5406	0.6724	0.5608
PSNR	18.1434	11.8587	16.797	17.1514	17.5293
